# Posterior Detachment of the Anorectum Following Blunt Trauma to the Perineum

**DOI:** 10.7759/cureus.19389

**Published:** 2021-11-09

**Authors:** Meetham Allawati, Murtadha Al Qubtan, Tagalsir Logman, Mohammed F Bondre, Abdullah Al Lawati, Lubna S Al Hashmi, Noor Al-Wahaibi, Osama Al-Lawati

**Affiliations:** 1 General Surgery, Royal Hospital, Muscat, OMN; 2 College of Medicine and Health Sciences, Sultan Qaboos University, Muscat, OMN; 3 College of Medicine, Trinity College Dublin, Dublin, IRL

**Keywords:** surgery, anorectum, case report, perineum, trauma

## Abstract

To the best of our knowledge, there have been few reports in the literature about perineal injury without an associated pelvic fracture. In this report, we are going to discuss the mechanism, management, and outcome of two cases of perineal injury associated with car accidents.

The two cases, one male and one female, presented with sustained isolated soft tissue injuries to the perineum. Both cases revealed a separated anorectum from the coccyx and sacrum without detectable damage to the lumen or surrounding sphincter. Pelvis fracture was not present in either case nor did the urinary bladder or urethra show signs of injury. A defunctioning (temporary) colostomy medical innervation was done to both and the perineal wound was left to heal. At last, both patients were satisfied with the final outcome.

## Introduction

The perineum is the diamond-shaped space located at the pelvic outlet, bordered by the pubic symphysis anteriorly and coccyx posteriorly. The levator ani and coccygeus muscles are present within it [1]. Injuries to the perineum and lower genitourinary tract are often acute. A study concludes that penetrating injuries such as gun wounds and knife stabs accounted for 35% of cases, and blunt trauma associated with falls and car accidents accounted for the remaining 65% [2]. The cases presented in this report are of the blunt trauma type. Blunt trauma perineal injuries are very rare with an incidence of 0.1% and a male to female ratio of 85:15 [3]. According to a study done by Teixeira et al., the overall mortality of the blunt trauma type was found to be 36%, demonstrating the severity of such incidences [4]. Pelvic fractures are often associated with blunt perineal injuries with their repair requiring complex procedures [5]. Diagnosis of blunt perineal traumas is variable depending on the affected structures, and their treatment requires a multidisciplinary approach, which can include procedures such as colostomies, diversions with bladder catheters, suprapubic cystotomies, and nephrostomies [6]. In this report, we present two cases of blunt trauma that caused perineal injuries with no fractures.

## Case presentation

Case 1

A 29-year-old male was brought to the emergency department following a car accident. It was reported that the wheel of a 5-ton water tanker rolled over his lower body as he was stepping into a car. Workup revealed a Glasgow Coma Scale (GCS) score of 15 with a pulse rate of 80 beats per minute and blood pressure of 90/60 mmHg. There was profuse bleeding due to a perineum injury. No fractures of the skull, cervical spine, and pelvis were detected in the X-rays. Urinary catheterization was performed without difficulty and there was no hematuria. No internal bleeding was seen during the abdominal ultrasound. The patient was immediately taken to the operating theatre for injury assessment after the replacement of fluid and blood.

Operative Findings and Procedure

While the patient was placed in the lithotomy position, the anterior displacement of the anus could be seen with a circular skin defect surrounding the region. The anus looked normal. There was a remaining 1 cm of normal perianal skin with no actual skin loss. Digital and proctoscopy examination showed an intact anorectum and its surrounding sphincters. Whereas exploring the wound revealed complete disruption of the posterior pelvic floor with lateral extensions into both gluteal regions and the right thigh. In addition, a hand could be passed behind the rectum and into the sacral promontory.

**Figure 1:**
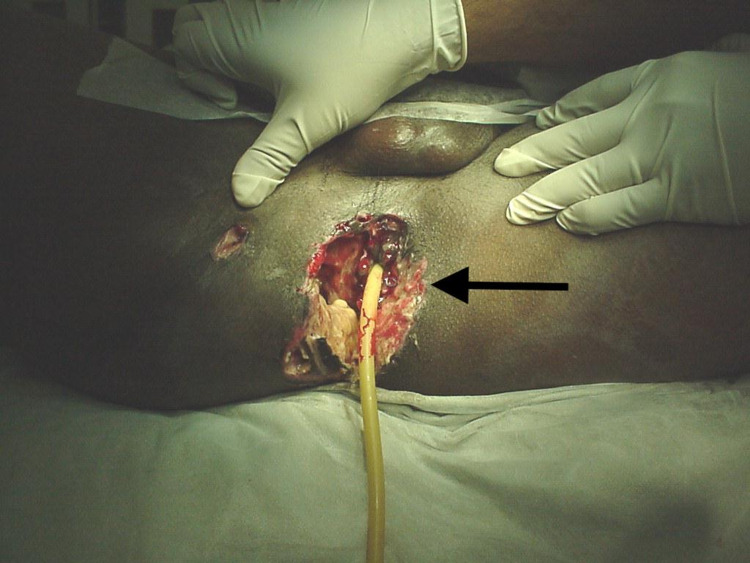
Site of injury of Case 1 before treatment.

**Figure 2:**
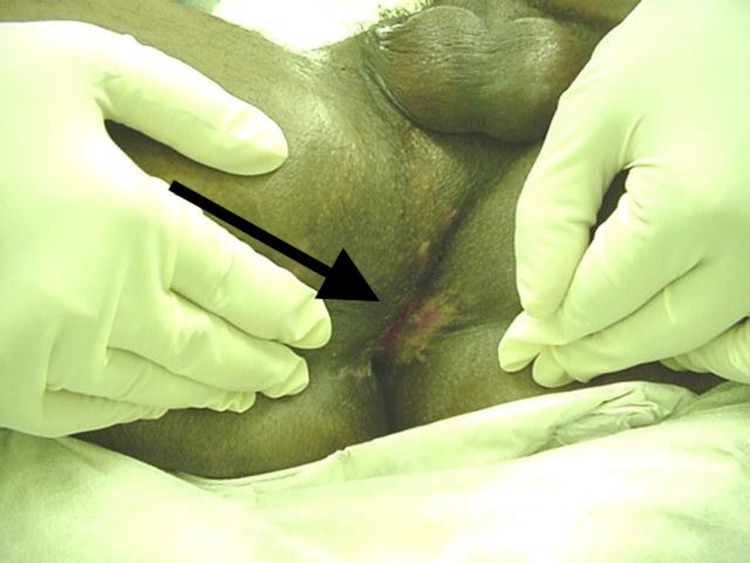
Site of injury of Case 1 after treatment.

The case was associated with extensive venous bleeding with the absence of major arterial tears. It was managed by the insertion of multiple packs along with partial closure of the skin defect. A small incision was made to perform a defunctioning sigmoid colostomy. No intra-abdominal bleeding was reported. The packs were removed two days later without any bleeding complications. A total of 22 units of blood were required during the first 48 hours of admission. Figures 1, 2 show the site of injury before and after treatment, respectively.

Progress

Continuous follow-up examinations were done during the following months of the accident. Seven examinations under anesthesia were performed. The skin was not closed completely at the site of the procedure. Consequently, it was decided not to reallocate the anorectum to a further posterior direction and the wound was left open. Initially, infection of deep injury recess was found and debridement and daily cleaning with betadine was performed. In later stages, as the wound became clean, saline dressings were substituted and the wound healed by granulation.

The defect was reduced to an area of 4 x 2 cm after four months of the procedure and was contracted towards the coccyx. Anterior to this, the anus felt normal on digital examination with a good sphincter tone and squeeze.

Physiological tests were done to test the sphincter function and sensation. Manometry showed the resting sphincter pressure of around 30 cmH2O. This increase in squeeze pressure was due to a combination of contraction of the puborectalis and gluteal muscles. The presence of rectal sensation was noted but not quantified. Defecography was assessed using a defecation proctogram. It revealed a normal anorectum that was displaced towards the pubic bone. Furthermore, although he was able to evacuate barium paste from the rectum, the anorectal angle did not widen during straining at the angle of 90°. Anyhow, there was no abnormal descent of the rectum on straining.

Five months after the surgical procedure, the perineal wound healed, and the colostomy was closed. The patient was able to pursue normal defecation without any episodes of major or minor incontinence. In the following follow-up visit, the patient expressed satisfactory defecation and micturition without incontinence.

Case 2

A 44-year-old female patient had a car accident and she was thrown out from the car landing on a hard rock on her buttocks. In the district hospital, she was found to have a partial laceration of the posterior perineum surrounding an intact anorectum. The bladder, urethra, and pelvis were not injured. A sigmoid colostomy was performed and then she was transferred to a tertiary hospital.

She had a crescent-shaped wound surrounding the anus from the three to nine o’clock position. In addition, the anus was displaced forward towards the vagina. Digital and proctoscopy examinations showed an intact anorectum and surrounding sphincters.

Progress

In this case, the patient's wound was subject to identical conservative management procedures as in the previously mentioned case. However, the patient was discharged from the hospital earlier as it was determined that she had adequate support from a daughter who has adequate experience in nursing. As the patient's wound was clean and in its proliferation stage of healing, the patient was instructed to perform daily irrigation using handheld bidets. She was scheduled for follow-up appointments every three weeks, which she attended punctually. Consequently, the proper management of the wound caused it to be superficial and reduced to 1 x 2 cm after five months of the procedure, with no infections.

The defecation portogram performed in a follow-up appointment revealed an anteriorly displaced anus with an anorectal angle of 70°; this angle only widened to 90° on straining, and while evacuation occurred, it was incomplete. However, no atypical rectum descent was noticed, and the patient reported complete evacuation in the toilet after the examination.

Seven months post-injury, the colostomy was closed, and five days after stoma closure, the patient reported normal bowel movement with no incontinence experienced and was subsequently discharged.

**Figure 3:**
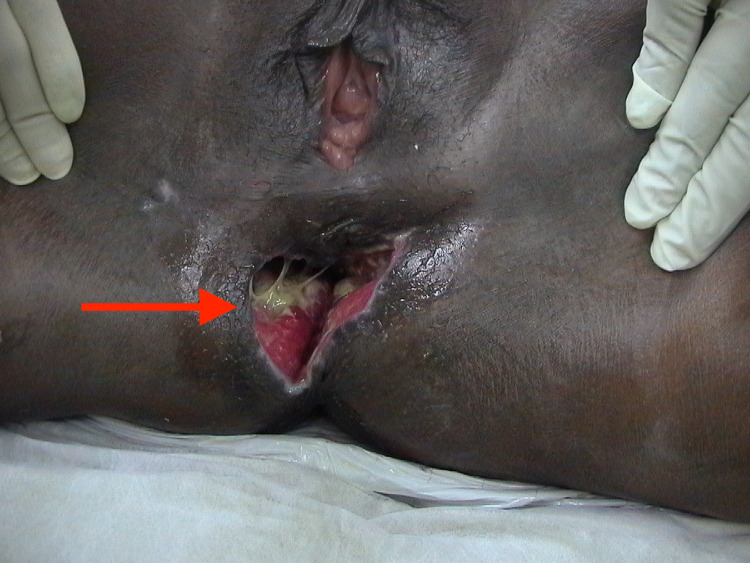
Site of injury of Case 2 before treatment.

**Figure 4:**
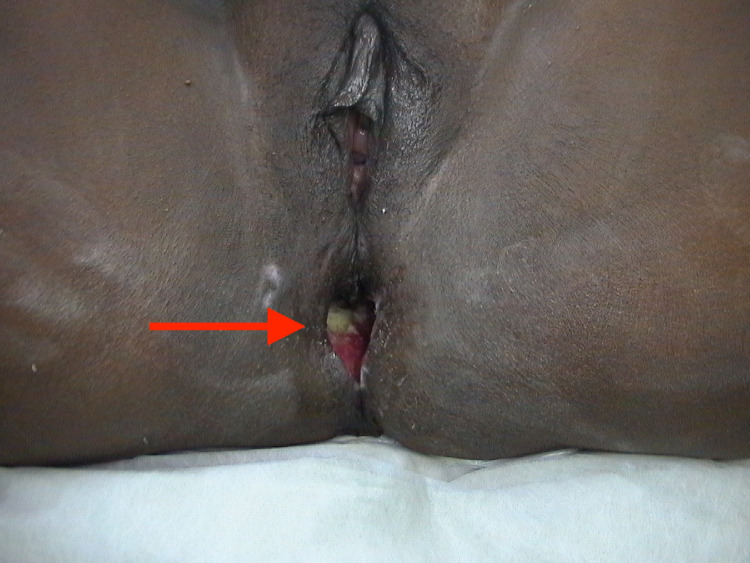
Site of injury of Case 2 after treatment.

Fifteen months post-injury, a follow-up report noted complete healing of the wound with adequate defecation. However, the patient reported the occasional need to use glycerin suppositories. Moreover, the patient's regime of treatment resulted in there being no fecal impaction. Figures 3, 4 show the site of injury before and after treatment, respectively.

## Discussion

Extent of injuries

In both cases, the severity of the injuries can be explained by superficial perineal skin tears and profound damage to the underlying muscles. Both cases exhibited superficial injuries sustained by skin with no actual skin loss and anterior displacement of anus towards the pubic area. In the first case, perineal skin was wholly detached from the remaining skin surrounding the anus. This detachment led to the isolation of the anus as it was contracted deeply to be surrounded by the subcutaneous fat layer. In the second case, skin separation exhibited a crescent-shaped laceration on the posterior end of the perineum. This left the anus anteriorly connected to the skin of the perineal body.

The deep injuries in both cases can be described as the detachment of the pubococcygeus, the iliococcygeus, and the coccygeus muscles from their distal attachments. Their detachment from the coccyx, in both cases, led to the anterior displacement of the anus as the anococcygeal raphe and the puborectalis muscle act as spring that contracts the anorectum anteriorly toward the pubis. In addition, the venous hemorrhage exhibited in both cases can be attributed to the blunt force trauma experienced by the two patients caused the stripping of the rectum for the presacral fascia, which led to an injury of fascia and the underlying veins supplying the perineum. Furthermore, the first patient suffered from additional separations into the right and left gluteal regions. He particularly endured further detachments into his right thigh for approximately 10-15 centimeters. No muscle damage was cited in these areas, and it was determined that the extent of his injury involved the dissociation of interfacial planes and the disruption of venous drainage. In addition, a detachment of the levator ani from its insertion into the obturator internus fascia was determined to an unknown extent. However, it was determined that this separation caused an opening of the retro rectal space to the level of the pelvic peritoneum. It was undetermined whether the splanchnic nerves sustained any damages in both cases.

Interestingly, both cases cited the absence of internal organ injury and the absence of bony pelvic fractures.

Mechanism of injury

As per the patient’s description in Case 1, his injury was crushing to the lower part of his body due to the double rears passing over him. This outcome could have been much worse had this happened across the front or side of the patient’s pelvis, as it would have led to the compression of his ileum and would have most likely resulted in fractures. On the other hand, had he been lying face down, the soft tissue of the patient’s buttocks and perineum might have absorbed the weight of the vehicle. This would’ve resulted in the compression of the anus and levator ani up towards the abdomen, and with the pressure release, the pelvic diaphragm would have possibly returned to its position rapidly with considerable negative pressure. The explanation regarding the separation of soft tissue is that it was a result of either the initial compression or of the sudden decompression.

In Case 2, the patient stated that her buttock landed squarely on top of a rock as she was thrown from the car. Due to her moderate obese nature (>100 kg), this fall may have compressed the pelvic diaphragm in the same way as Case 1 while at the same time the pelvic bones would have been protected from a fracture as the soft tissues absorbed the energy of the fall. Therefore, it is safe to assume that the tissue separation, in this case, was an outcome of compression and not decompression.

Plan of management

A visual and manual examination was done to assess the extent of disruption to the pelvic floor in both cases. There was no identification of any tissue that could have been repaired. In Case 1, the bleeding was so extensive that packing and control was the first and most important thing to be done. There were attempts to reposition the anus and close the skin defect; however, they were unsuccessful. Due to the fact that the internal and external sphincters were intact, as well as the fact that both the puborectalis muscle and the anorectal tube were not disrupted, a conservative management plan was set for both cases. Sigmoid loop colostomy was performed for fecal diversion and there were no attempts to repair the disrupted muscle or to relocate the anus posteriorly. The skin was left open, accompanied by daily dressings until the wound closed. This was done in a hospital for the patient in Case 1 as it was a large initially infected wound. Since the wound was clean in Case 2, smaller daily irrigation with water at home was both effective and sufficient.

Assessment of the final result

There was a return to normal rectal evacuation without incontinence. Both patients (at two years and 15 months) were satisfied with the results. Continence of feces, mucus, and gas was present, thanks to the sphincter mechanism and puborectalis sling being intact, despite the fact that they were anatomically displaced. No current information about sexual function, in either case, is present; however, the first patient got married and conceived a child six months after his injury. The second patient is a widow. The urinary bladder and voiding mechanism functioned normally in both cases.

In the long term, there is a possibility of perineal hernia, rectal intussusception, or prolapse; however, no such evidence has been presented as of now.

## Conclusions

The present report describes an approach to cases with traumatic injuries to the perineum. It is important to know in depth the anatomy of the perineum and its organs, as well as the implications of the hemodynamic stability of the patient when deciding the appropriate method of treating the injuries. Patients who present with blunt soft injury of the perineum without any visceral or bony damage can be managed by controlling the bleeding, fecal diversion, and allowing the open wound to heal by simple dressing or cleansing. Return to full continence is expected after four to six months from the day of the accident.

## References

[1] Choe J, Wortman JR, Sodickson AD, Khurana B, Uyeda JW (2018). Imaging of acute conditions of the perineum. Radiographics.

[2] Paparel P, N'Diaye A, Laumon B, Caillot JL, Perrin P, Ruffion A (2006). The epidemiology of trauma of the genitourinary system after traffic accidents: analysis of a register of over 43,000 victims. BJU Int.

[3] Petrone P, Inaba K, Wasserberg N (2009). Perineal injuries at a large urban trauma center: injury patterns and outcomes. Am Surg.

[4] Teixeira FJR Jr, do Couto Netto SD, Collete e Silva FS (2015). Complex perineal injuries in blunt trauma patients: the value of a damage control approach. Panam J Trauma Crit Care Emerg Surg.

[5] Wei R, Cao X, Tu D (2012). Clinical treatment of open pelvic fractures associated with perineal injury. (Article in Chinese). Zhongguo Xiu Fu Chong Jian Wai Ke Za Zhi.

[6] Petrone P, Rodríguez Velandia W, Dziaková J, Marini CP (2016). Treatment of complex perineal trauma. A review of the literature. Cir Esp.

